# LRP6 is identified as a potential prognostic marker for oral squamous cell carcinoma via MALDI-IMS

**DOI:** 10.1038/cddis.2017.433

**Published:** 2017-09-07

**Authors:** Yao Yuan, Xiaoyan Xie, Yuchen Jiang, Zihao Wei, Peiqi Wang, Fangman Chen, Xinyi Li, Chongkui Sun, Hang Zhao, Xin Zeng, Lu Jiang, Yu Zhou, Hongxia Dan, Mingye Feng, Rui Liu, Zhiyong Wang, Qianming Chen

**Affiliations:** 1 State Key Laboratory of Oral Diseases, National Clinical Research Center for Oral Diseases, West China Hospital of Stomatology, Sichuan University, Chengdu, China; 2 Department of Stomatology, The Second Xiangya Hospital, Central South University, Changsha, China

## Abstract

Oral squamous cell carcinoma (OSCC) is a leading cause of cancer-related deaths worldwide, with 500 000 new cases each year. However, the mechanisms underlying OSCC development are relatively unknown. In this study, matrix-assisted laser desorption ionization imaging mass spectrometry (MALDI-IMS)-based proteomic strategy was used to profile the differentially expressed peptides/proteins between OSCC tissues and their adjacent noncancerous tissues. Sixty-seven unique peptide peaks and five distinct proteins were identified with changed expression levels. Among them, LRP6 expression was found to be upregulated in OSCC tissues, and correlated with a cluster of clinicopathologic parameters, including smoking, drinking, tumor differentiation status, lymph node metastasis and survival time. Notably, knockdown of LRP6 inhibited the proliferation ability of OSCC cells. Furthermore, we demonstrated that the expression of LRP6 in OSCC cells is positively correlated with its downstream oncogene, *FGF8*. The present study suggests that LRP6 could be a potential biomarker for OSCC patients, and might further assist in the therapeutic decisions in OSCC treatment.

Oral cancer is one of the most common cancers worldwide, and over 90% of oral cancers are oral squamous cell carcinomas (OSCCs).^1^ Despite the advanced therapeutic strategies applied in treating OSCC patients during recent years,^2, 3^ only limited improvement was achieved in the overall prognosis of this disease, and the 5-year survival rate is still below 50%.^4, 5^ Therefore, a better understanding of OSCC etiopathogenesis, especially the molecular determinants in OSCC prognosis and valuable biomarkers, is still needed.

Proteomics stands for a concept of an entire set of proteins expressed by a whole genome, which fills the gap between cell function and the information encoded by genome. Proteomics approaches are powerful tools in screening molecules in either clinical or experimental samples, and have been widely applied in cancer research. Matrix-assisted laser desorption ionization imaging mass spectrometry (MALDI-IMS) is an emerging technique that allows profiling up to hundreds of molecules directly from a tissue section or tissue array.^6^ One of the major advantages of this technology lies in that it is capable of measuring both the abundance and distribution of the entire proteome throughout a tissue section, without any artificial labeling or modification.^7^ Although possessing 'profiling' property, other proteomic approaches, such as two-dimensional gel electrophoresis or liquid chromatography LC-MS/MS, are incapable of analyzing the spatial distribution of peptides/proteins in the histological context.^8^ MALDI-IMS has been used in many proteomics-related researches.^9, 10^ Thus far, a small but increasing number of studies have used MALDI-IMS in screening the abnormally expressed peptides/proteins in diverse cancers, including brain cancer,^11^ prostate cancer,^12^ lung cancer^13^ and head and neck cancer,^14^ and a cluster of identified peptides/proteins were shown as potential biomarkers. In this study, the MALDI-IMS-based proteomic strategy is used to compare protein expression patterns between OSCC tissues and adjacent noncancerous counterparts. LRP6 is found to be highly expressed in OSCC areas, and is associated with an index of histopathological parameters. Further, we demonstrate that a combination of LRP6 and its downstream protein, FGF8, could be a potential prognostic factor for OSCC outcome.

## Results

### MALDI imaging

**Figure 1:**
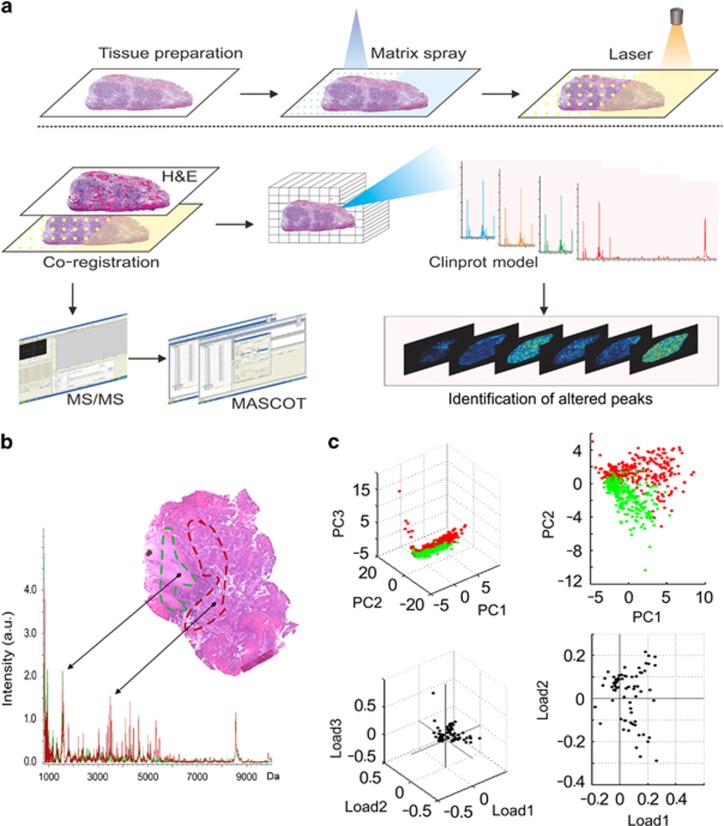
MALDI-IMS analyses of OSCC and adjacent noncancerous areas. (**a**) Schematic illustration of the workflow of MALDI-IMS analyses of OSCC tissues. (**b**) Representative MALDI spectrums and hematoxylin and eosin (H&E) staining imaging of OSCC areas (red) and adjacent noncancerous areas (green). (**c**) Principal component analysis (PCA) analyses were performed to examine the multidimensional distributions of the identified peaks detected from both OSCC (red) and noncancerous (green) areas. a.u., Arbitrary unit

**Figure 2:**
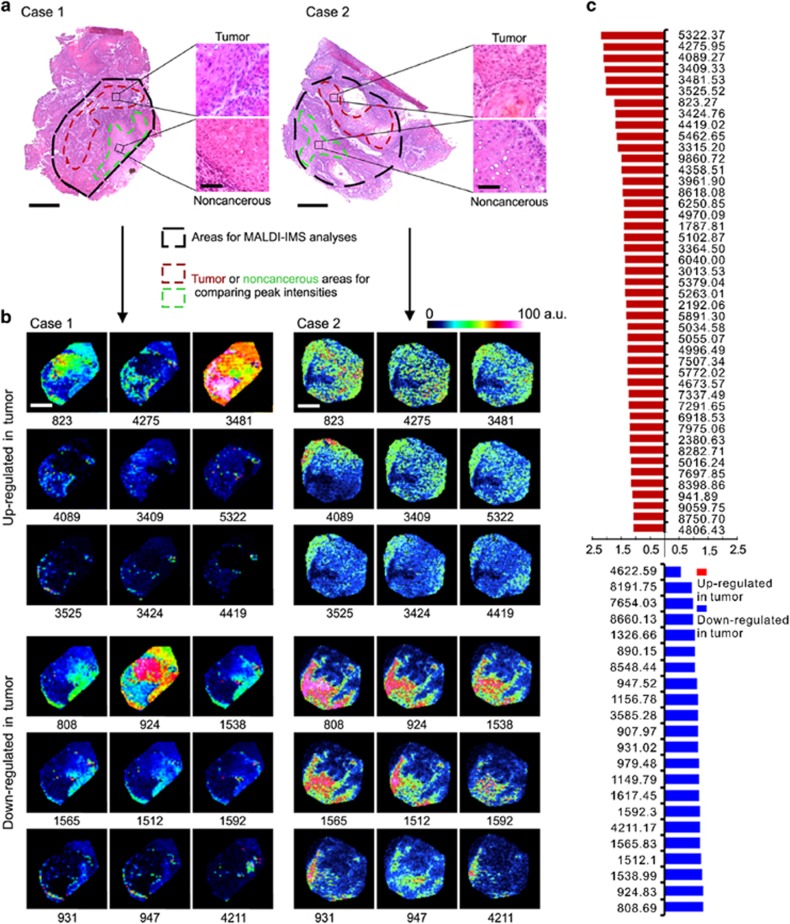
Altered peptide peaks identified by MALDI-IMS. (**a**) Representative hematoxylin and eosin (H&E) staining images for OSCC areas and the adjacent noncancerous areas. The areas used for MALDI-IMS analyses were labeled by black dotted lines. The OSCC and adjacent noncancerous areas selected for identifying altered peaks were labeled by dark red or light green dotted line, respectively. Scale bar: original images, 250 *μ*m; enlarged images, 50 *μ*m. (**b**) Representative MALDI-MS images of 18 peaks with most significant alterations. Sample cases 1 and 2 are the same samples as shown in (**a**). The corresponding *m/z* value of each peak was shown under the image. (**c**) Intensities of 67 altered peaks. a.u., Arbitrary unit

To determine OSCC-specific peptide/protein expression patterns, 10 OSCC clinical tissues, containing both cancer and adjacent noncancerous region, were subjected to MALDI-IMS analyses. The schematic flow diagram of MALDI-IMS analyses was shown in Figure 1a. The representative H&E staining images indicating the areas for MALDI-IMS analyses, and the tumor or non-tumor areas for comparing peak intensities, was shown in Figure 2a. Representative MS spectrums from OSCC (red) and adjacent noncancerous (green) areas were shown in Figure 1b. By principal component analysis, each peak was calculated and converted into a unique point in a two- or three-dimensional coordinate system. As shown in Figure 1c, the points representing the peaks from OSCC areas gathered in a separated cluster, in contrast to those points representing the peaks from adjacent noncancerous areas, suggesting histological heterogeneity of the identified peaks. Further, normalization of each peak was performed according to the average ionic intensity, and the changed peaks between OSCC and control areas were subsequently calculated by using the ClinprotTools 3.0 Software (Bruker Daltonics, Ettlingen, Germany). As listed in Supplementary Table S1, 45 peaks were upregulated in OSCC areas (*P*<0.01), whereas 22 peaks were downregulated in cancerous areas (*P*<0.01). MALDI-IMS images of 18 peaks with most discriminative expression (9 upregulated and 9 downregulated in OSCC areas) were shown in Figure 2b, respectively. Additionally, altered levels of 67 peaks were shown in Figure 2c.

### Protein identification

Out of the 67 identified peaks, 5 peaks with relative higher signal/noise value and intensity were manually selected for protein identification. The original MS/MS results were submitted to and analyzed by Mascot service and/or ExPASy protein sequence database. As a result, five distinct proteins were positively identified, including S27A3, LRP6, MKKS, DOCK9 and HXA2, corresponding to peak 808.69, 823.27, 924.83, 1538.99 and 3409.33, respectively. Detailed information of these identified proteins, including Uniprot access number, peptide sequence and protein description, were provided in Table 1.

### Bioinformatics analysis

**Figure 3:**
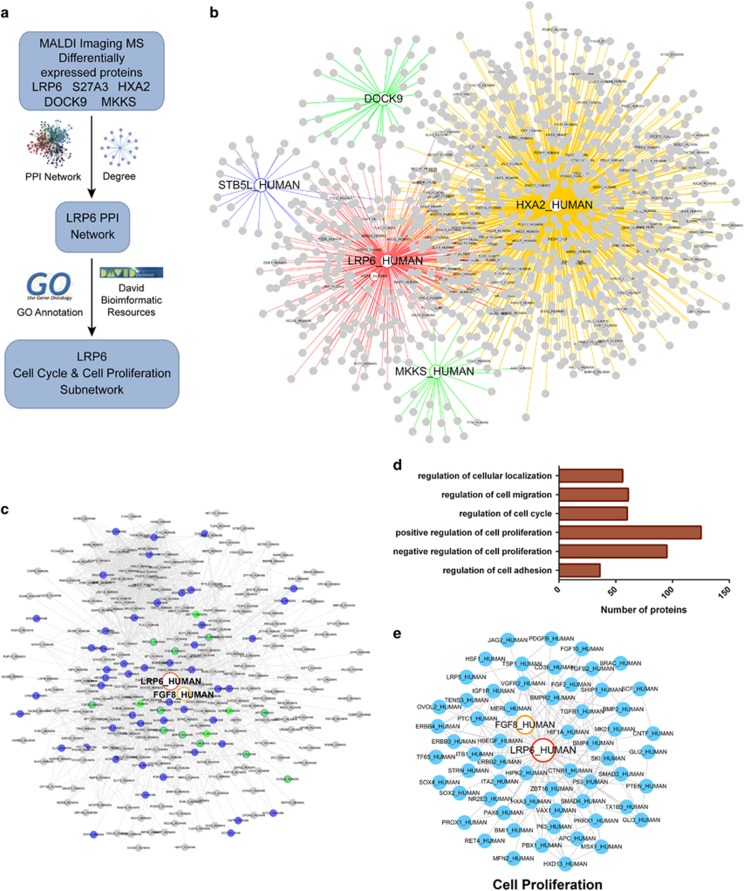
Bioinformatics analysis of proteins identified from MALDI-IMS. (**a**) Schematic illustration of the workflow of bioinformatics analyses. (**b**) Total PPI network was established for the five proteins identified from MALDI-IMS. (**c**) Sub-PPI network for LRP6-related proteins was extracted from the total network. (**d**) Predicted LRP6-related proteins were divided into several groups based on their function. (**e**) Predicted LRP6-related proteins involved in the regulation of cell proliferation

To investigate the potential roles of the five identified proteins in OSCC development, protein–protein interaction (PPI) network with functional annotations was further established (Figure 3a). As shown in Figure 3b, a total of 1069 paired PPIs were extracted from the pre-PPI network, which was built up based on the predicted proteins that correlated with the five identified proteins. The PPI network was further processed by GO annotation functional cluster analysis. Strikingly, a significant proportion of LRP6-associated proteins were found in the regulation of cell proliferation (Figures 3c–e). By gene ontology (GO) annotation, the *P*-value for genes in the regulation of cell proliferation is 5.2E−19. Particularly, the *P-*value for genes in a positive regulation of cell proliferation is 1.2E−67, and the *P*-value for genes in a negative regulation of cell proliferation is 8.5E−50. Considering that the unlimited cell proliferation is an important feature of cancer cells, thus *LRP6* was chosen for further studies.

### LRP6 expression is correlated with OSCC development

**Figure 4:**
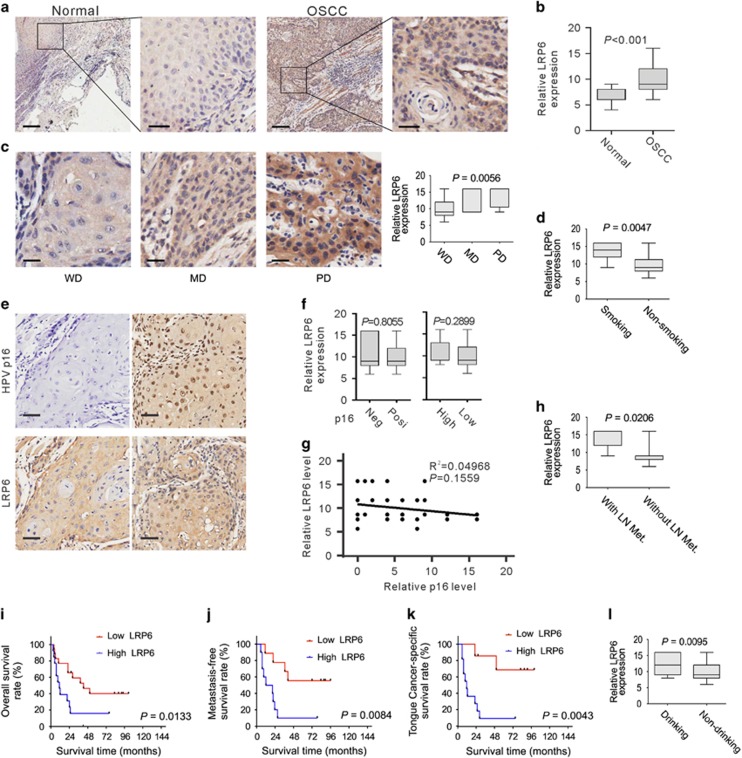
LRP6 upregulation is correlated with OSCC development. (**a**) Representative images of LRP6 immunostaining of OSCC tissues and normal oral mucous tissues. Scale bar: left panels, 500 *μ*m; right panels, 100 *μ*m. (**b**) LRP6 immunostaining scores in OSCC tissues and normal oral mucous tissues were analyzed. (**c**) LRP6 immunostaining scores in well, moderately or poorly differentiated tumors were analyzed. WD, well differentiated; MD, moderately differentiated; PD, poorly differentiated. (**d**) LRP6 immunostaining scores in OSCC patients with or without smoking were analyzed. (**e**) The representative p16-negative or -positive staining results and the corresponding LRP6 staining results. (**f**) Left: the relative LRP6 expression level in HPV p16-positive or -negative OSCC tissues was compared. Right: the relative LRP6 expression level in OSCC tissues with high or low p16 expression was compared. (**g**) Pearson's test was used to analyze the correlations between the LRP6 and HPV P16 expression level. (**h**) LRP6 immunostaining scores in tumors with or without lymph node metastasis were analyzed. LN Met, lymph node metastasis. (**i**) Overall survival time of OSCC patients with high or low LRP6 expression was analyzed by Kaplan–Meier analysis. (**j**) LRP6 immunostaining scores in OSCC patients with or without drinking were analyzed. (**k**) Survival time of tongue cancer patients with high or low LRP6 expression was analyzed. (**l**) Survival time of metastasis-free OSCC patients with high or low LRP6 expression was analyzed

To extend our findings in MALDI-IMS and bioinformatics analysis, the expression of LRP6 in OSCC and normal oral mucous tissues was examined by immunostaining. Clinicopathologic information of clinical samples was summarized in Supplementary Table S2. As shown in Figure 4a, LRP6 signal was positively detected in both the cytoplasm and membrane, which were consistent with previous studies.^15^ Notably, strong LRP6 immunoreactivity was found in most tumor cases, whereas most of the cases of normal mucous exhibited only weak staining of LRP6 (*t*-test; OSCC *N*=51, normal *N*=28; *P*<0.001; Figure 4b).

Next, we sought to evaluate the relevance between LRP6 expression and a series of clinicopathologic factors in OSCC samples. We found that the level of LRP6 expression was positively associated with smoking (*t*-test; with smoking *N*=8, without smoking *N*=20; *P*=0.0047; Supplementary Table S2 and Figure 4d) and drinking (*t*-test; with drinking *N*=13, without drinking *N*=15; *P*=0.0095; Supplementary Table S2 and Figure 4l). Further, LRP6 immunoreactivity was more intense in tumor with lymph node metastasis (*t*-test; with node metastasis *N*=9, without node metastasis *N*=19; *P*=0.0206; Supplementary Table S2 and Figure 4h). In contrast to well-differentiated tumor, a higher level of LRP6 was found to be in the poorly and moderately differentiated (ANOVA; well differentiated 18, moderately differentiated 5, poorly differentiated 5; *P*=0.0056; Supplementary Table S2 and Figure 4c). However, no apparent correlation was observed between LRP6 expression and patient gender, age, tumor size, tumor location or clinical stage (Supplementary Table S2). To evaluate the relations between LRP6 expression and the HPV status, HPV p16 IHC staining was performed. HPV p16 expression was defined based on IHC staining score: score=0, negative; score >0, positive; score ⩽8, low; score >9, high. As shown in Figures 4e and f, no obvious difference in LRP6 expression was found between p16-positive and -negative samples, or between the sample with high or low p16 expression. Further, statistical analyses showed that the expression of HPV p16 is not correlated with LRP6 (*P*=0.1559, *R*^2^=0.04968; Figure 4g). These data suggest that LRP6 expression is not associated with HPV infection status in OSCCs.

Further, the Kaplan–Meier method and log-rank test were used to estimate the regulatory role of LRP6 expression on the survival rates of OSCC patients. As a result, those patients with high LRP6 expression level showed significantly decreased average survival time after surgery than those patients with low LRP6 expression (Figure 4i). Further, a shortened metastasis-free survival time is more likely to be associated with patients with high LRP6 expression (Figure 4j). The relationship between LRP6 expression and the outcome of tongue cancer patients was also examined, as tongue cancer was ranked as the most common subtype of OSCCs.^16^ As shown in Figure 4k, LRP6 expression was negatively associated with survival time of tongue cancer patients. These results suggested that LRP6 was upregulated in OSCCs, and is negatively correlated with patient outcome.

### Modulation of LRP6 regulates OSCC cell proliferation

**Figure 5:**
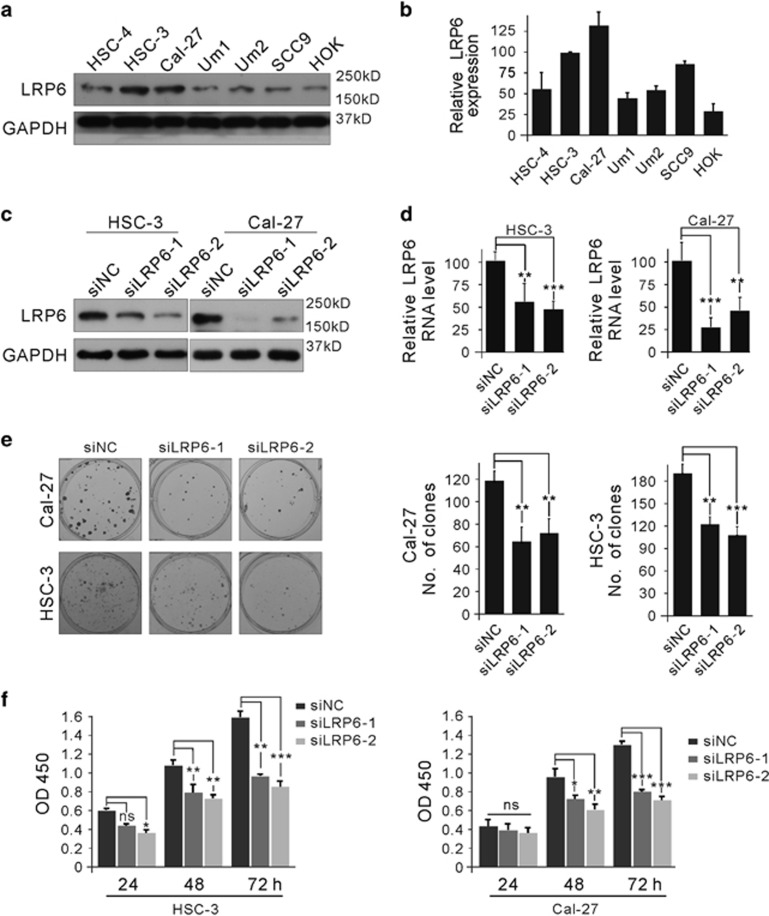
Expression level of LRP6 was associated with the proliferation ability of OSCC cells. (**a**) Expression of LRP6 in normal oral squamous cell line and several OSCC cell lines was examined by immunoblot. (**b**) The relative mRNA level of LRP6 in these cell lines was examined by quantitative PCR (Q-PCR). (**c** and **d**) HSC-3 and Cal-27 cell lines were transfected with LRP6-specific small interfering RNAs (siRNAs), and the protein level was detected via immunoblot (**c**) or Q-PCR (**d**). (**e**) HSC-3 and Cal-27 cell lines were transfected with LRP6-specific siRNAs and cell proliferation was detected via the colony formation assay. (**f**) HSC-3 and Cal-27 cell lines were transfected with LRP6-specific siRNAs, and proliferation of HSC-3 and Cal-27 cell lines was detected by the Cell Counting Kit-8 (CCK8) assay

It has been demonstrated that LRP6 is involved in regulating proliferation in cancer cells.^17^ As a pilot test, LRP6 expressions in one normal oral squamous cell line (HOK) and six human OSCC cell lines (HSC-4, HSC-3, Cal-27, Um1, Um2, SCC9) were examined. As shown in Figures 5a and b, LRP6 was highly expressed in HSC-3 and Cal-27 cell lines both at protein and RNA levels, and the LRP6 expression was relatively low in HOK, HSC-4, Um1, Um2 and SCC9 cell lines. Therefore, HSC-3 and Cal-27 cell lines were selected as *in vitro* cell models. To avoid off-target effects, two siRNAs targeting different sites of LRP6 were designed. As shown in Figures 5c and d, each siRNA could efficiently reduce the LRP6 expression levels in both two cell lines. Notably, knockdown of LRP6 by either siLRP6 substantially reduced the proliferation rate of HSC-3 and Cal-27 cells (Figure 5e), revealed by the colony formation assay. Similar proliferation inhibitory effects were observed from the CCK8 assay (Figure 5f). These results suggested that LRP6 possessed a proproliferative property in OSCC cell lines.

### LRP6 expression is positively correlated with proto-oncogenic protein FGF8

**Figure 6:**
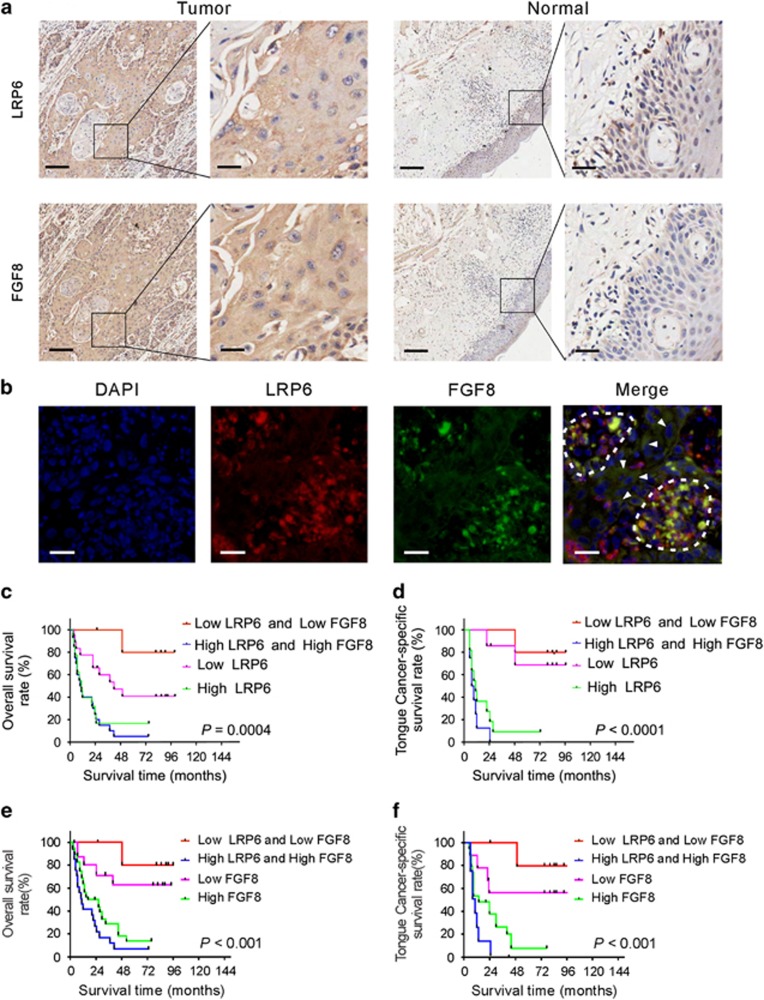
Expression of LRP6 is positively associated with proto-oncogenic protein FGF8. (**a**) Representative images of LRP6 and FGF8 immunostaining in OSCC tissues and normal oral mucous tissues using sister slides. Scale bar: left panels, 500 *μ*m; right panels, 100 *μ*m. (**b**) Immunofluorescent staining of FGF8 (green) and LRP6 (red) in OSCC tissues. The cells with both high FGF8 and LRP6 expression are circled, and the arrowheads indicate the cells with both low FGF8 and LRP6 expression. (**c**) Overall survival time of OSCC patients with high LRP6 expression alone, low LRP6 expression alone, concurrent high LRP6/FGF8 expression or concurrent low LRP/FGF8 expression was analyzed. (**d**) Survival time of tongue cancer patients with high LRP6 expression alone, low LRP6 expression alone, concurrent high LRP6/FGF8 expression and concurrent low LRP/FGF8 expression was analyzed. (**e**) Overall survival time of OSCC patients with high FGF8 expression alone, low FGF8 expression alone, concurrent high LRP6/FGF8 expression or concurrent low LRP/FGF8 expression was analyzed. (**f**) Survival time of tongue cancer patients with high FGF8 expression alone, low FGF8 expression alone, concurrent high LRP6/FGF8 expression and concurrent low LRP/FGF8 expression was analyzed

Previously, we reported that FGF8 promoted cell proliferation in colorectal cancer cells.^18^ Considering that LRP6 is an essential Wnt coreceptor for activating the canonical Wnt/*β*-catenin signaling pathway,^19^ and FGF8, which was found in the LRP6-associated PPI network (Figure 3e), was a potential downstream gene of the Wnt pathway,^20^ the regulatory role of LRP6 on FGF8 expression in OSCC tissues was of particular interest. Then, the expression pattern of LRP6 and FGF8 was compared in OSCC samples. FGF8 immunostaining was performed using the sister tissue slides corresponding to those used for LRP6 immunostaining. As shown in Figure 6a, the immunostaining signals of FGF8 was well paralleled with LRP6 in OSCC tissues. To further confirm the concurrent expression of LRP6 and FGF8 in OSCC tissues, co-immunofluorescent staining was conducted. As shown in Figure 6b, strong FGF8 signal (green) was frequently found in those cells with strong LRP6 signal (red), suggesting that expression of FGF8 and LRP6 was positively related.

Next, we examined whether concurrent expression of LRP6 and FGF8 could be a better prognostic factor for OSCC patients than LRP6 or FGF8 expression alone. As shown in Figures 6c and e, the patients with high expression of both LRP6 and FGF8 showed even shorter overall survival time compared with those patients with high LRP6 or FGF8 expression alone. Likewise, the concurrently low LRP6 and FGF8 expressions are associated with a better survival rate compared with low LRP6 or FGF8 expression alone. Similar relationship between concurrent expression of LRP6/FGF8 and patient outcome was also observed in tongue cancer (Figures 6d and f).

### FGF8 is required for LRP6-induced proliferation in OSCC cell lines

**Figure 7:**
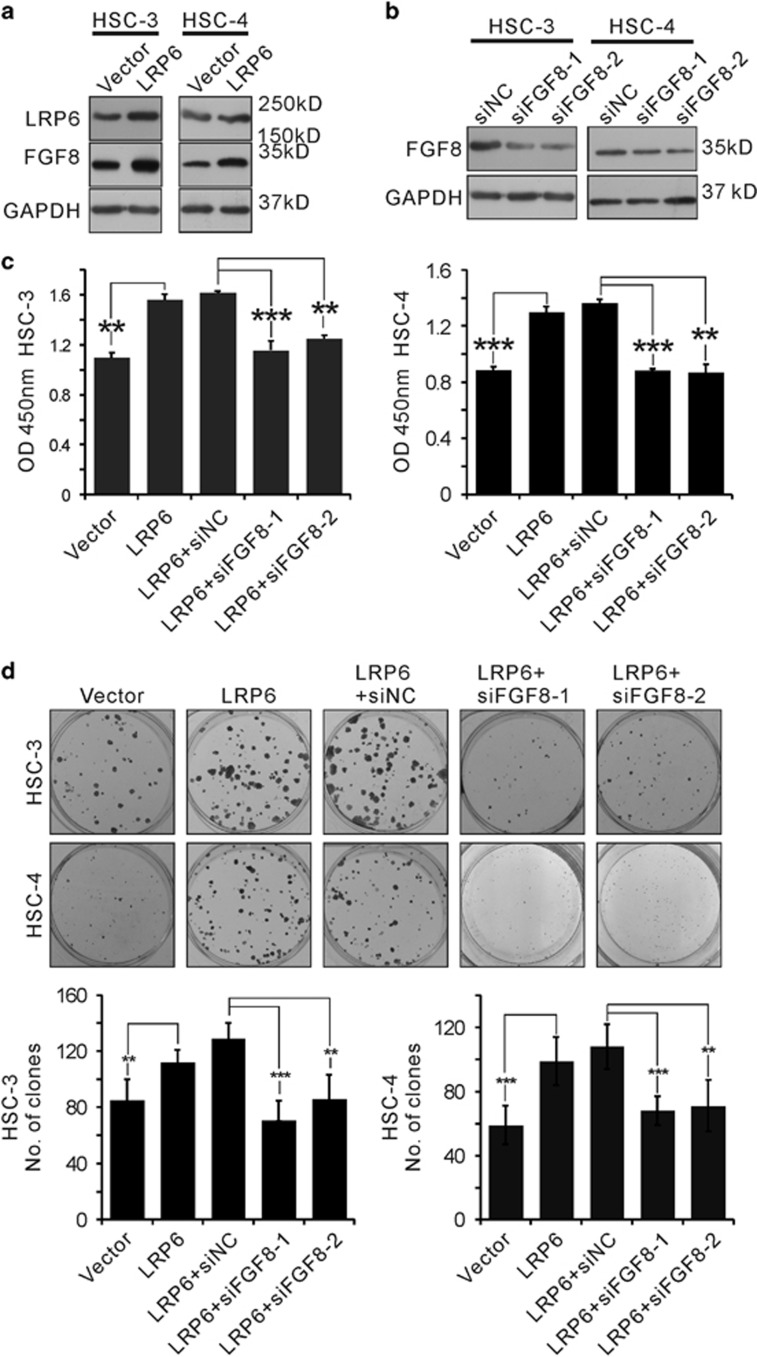
FGF8 is required for LRP6-induced proliferation in OSCC cell lines. (**a**) LRP6 expression plasmid was transfected into HSC-3 and HSC-4 cell lines. The expression level of LRP6 and FGF8 was detected via western blot (WB). (**b**) Two small interfering RNAs (siRNAs) targeting FGF8 were tested and the knockdown efficiencies were validated via WB. (**c** and **d**) HSC-3 and HSC-4 cell lines were transfected with LRP6 expression vector with or without FGF8 siRNA, and cell proliferation were detected by Cell Counting Kit-8 (CCK8) (**c**) and colony formation assays (**d**)

Next, we sought to determine whether FGF8 is required for LRP6-induced proliferation. As expected, overexpression of LRP6 triggered FGF8 transcription in both HSC-3 and HSC-4 cell lines (Figure 7a). Two distinct siRNAs targeting FGF8 was designed, and treatment with either siRNA markedly inhibited endogenous FGF8 expression (Figure 7b). Notably, knockdown of FGF8 by siRNAs substantially abolished LRP6-induced cell proliferation in both HSC-3 and HSC-4 cell lines, revealed by the CCK8 (Figure 7c) and colony formation assay (Figure 7d). These results suggest that FGF8 played an important role in LRP6-induced proliferation in OSCC cell lines.

## Discussion

OSCCs possess poor prognosis and strong potential metastasis, and the mortality rate of this disease is nearly 50% within 5 years. In spite of a large body of studies, the molecular mechanisms responsible for OSCC development remain unclear. Proteomic strategies have been widely applied as vital tools to screen potential diagnostic and prognostic biomarkers in human cancers. Compared with conventional proteomic technologies, MALDI image-based proteomics provide the spatial information of each detected signal across the tissue context.^21^ Further, disease-specific areas on the sample tissue slide can be precisely defined, according to an H&E-stained sister slide. These results have indicated that tumor or noncancerous areas were selected for comparing peak intensities, and these areas were defined according to H&E staining. Sixty-seven specific peptide peaks were detected with changed expression level in this study, including 45 peaks upregulated in OSCC areas and 22 peaks upregulated in noncancerous areas.

MALDI-IMS allows unbiased analysis of intact tissue sections, avoiding homogenization and separation steps and protecting the anatomical feathers *in situ*.^22^ MALDI-IMS has been applied in previous OSCC study; however, these studies failed in identifying proteins. In the present data, five proteins with altered expression levels, including S27A3, LRP6, MKKS, DOCK9 and HXA2, were successfully identified. The potential protein interaction network of each protein was addressed by bioinformatics analyses. PPI network with functional annotations was established and further processed by GO annotation cluster analysis. The bioinformatics analysis revealed that LRP6 was closely associated with the regulation of cell proliferation and cell cycle. Our data, together with previous reports,^23, 24^ suggest that MALDI-IMS combined with bioinformatics analyses is a powerful tool in identifying new cancer biomarkers.

It is documented that aberrantly increased LRP6 expression is involved in the development of several human cancer types, including breast cancer and prostate cancer.^25, 26^ Engineered overexpression of LRP6 was found to promote either cell proliferation or invasion in multiple *in vitro* or *in vivo* models.^27^ LRP6 expression level was validated via immunostaining, as the above results show that LRP6 was overexpressed in OSCC tissues. It has been reported that LRP6 was associated with diverse physiologic processes, including lipoprotein metabolism, protease regulation, glucose homeostasis, cell differentiation and cell migration.^28^ Our results show that LRP6 expression was strongly associated with lymph node metastasis and tumor differentiation status, as well as the habit of smoking and drinking. Notably, our results showed that the increased expression of LRP6 is associated with a shortened survival time, for either overall OSCC patients or tongue cancer patient subset.

LRP6 was previously involved in the regulation of cancer cells. It has been shown that the expression of LRP6 promote the proliferation rate of human fibrosarcoma HT1080 cells by altering the subcellular distribution of *β*-catenin.^29^ Another report revealed that the Wnt signaling was significantly activated in LRP6 transgenic mice, which contributed to the development of breast cancer.^30^ In this study, we showed that the proliferation rate of HSC-3 and Cal-27 OSCC cell lines were markedly inhibited after LRP6 knockdown, suggesting that LRP6 is probably a proproliferative factor in OSCCs.

It is widely accepted that LRP6 functions as essential coactivators for the canonical Wnt/*β*-catenin signaling.^19^ Wnt ligands interact with both frizzled and LRP6 to initiate canonical Wnt signaling.^31^ The binding of Wnt leads to phosphorylation of LRP6 coreceptor at cytoplasmic residues by glycogen synthase kinase-3 and casein kinase-1.^32^ Activated LRP6 recruits the scaffold protein axin to the membrane and prevents it from participation in the degradation of *β*-catenin, thereby enhancing translocation of *β*-catenin to the nucleus where it interacts with the LEF-1/TCF family of transcription factors to regulate transcription of Wnt target genes.^33^ Studies in chick and zebrafish have suggested that ectopic expression of Wnt1 can alter FGF8 expression through a pathway involving Lmx1b,^34^ Pax2 and En2.^20^ Additionally, Wnt/*β*-catenin signaling was reported to regulate isthmic FGF8 expression in mouse.^35^ These results suggested that FGF8 was a potential downstream gene of Wnt signaling, and our results indicated that LRP6 promote the expression of FGF8 in OSCC cells. In a previous study, we reported that activation of FGF8 contributed to metastasis and poor prognosis in patients with colorectal cancer. FGF8 can accelerate the growth rate, increase the clonogenic capability and induce an invasive phenotype in colorectal cancer cells, suggesting the proto-oncogenic property of FGF8.^18^ Here, by bioinformatics analyses, we found that FGF8 was present in LRP6-related PPI network. Indeed, overexpression of LRP6 triggered FGF8 expression in OSCC cell lines, and knockdown of FGF8 largely abolished LRP6-induced proliferation in OSCC cell lines. Interestingly, FGF8 expression is positively associated with LRP6 expression in OSCC clinical samples by immunostaining. More importantly, in contrast to LRP6 expression alone, our data show that concurrent expression of LRP6 and FGF8 could serve as a better factor to predict OSCC patient outcome.

## Materials and Methods

### Clinical samples

Ten OSCC specimens containing adjacent noncancerous areas for MALDI-IMS analysis and 20 normal oral mucous tissues for IHC analysis were collected from the Department of Oral and Maxillofacial Surgery, Hospital of Stomatology, Sichuan University. All the samples were obtained with informed consent of patients. This study was approved by the Institutional Ethics Committee of Sichuan University. The cancerous or noncancerous areas were identified by two pathologists independently, according to the HE staining. The pathologists were blinded to patient outcomes and other clinical information. If the evaluations did not agree, the sample were re-evaluated and then classified according to the assessment given most frequently by the pathologists.

## Publisher’s Note

Springer Nature remains neutral with regard to jurisdictional claims in published maps and institutional affiliations.

## Figures and Tables

**Table 1:** Proteins identified in MS/MS

**Peak no.**	***m/z***^a^	**Peptide sequence**^b^	**Uniprot no.**^c^	**Protein description**^c^	**Gene**^c^	**Mw**^d^	**pI**^d^	**Alteration in OSCC**^e^
1	808.69	MQLYTH	Q5K4L6	Long-chain fatty acid transport protein 3	*SLC27A3*	78.644	7.25	**↓**
2	823.27	SGSLPGM	O75581	Low-density lipoprotein receptor-related protein 6	*LRP6*	180.429	5.12	↑
3	924.83	IPVDFSS	Q9NPJ1	McKusick–Kaufman/Bardet–Biedl syndromes putative chaperonin	*MKKS*	62.341	6.67	**↓**
4	1538.99	R.IRT VLMATAQM K.E+oxidation (M); 2 phospho (ST)	Q9BZ29	Dedicator of cytokinesis protein 9	*DOCK9*	236.445	7.25	**↓**
5	3409.33	TSFPPVADTFQSSSIKTSTLSHSTLIP	O43364	Homeobox protein Hox-A2	*HOXA2*	41.001	5.54	↑

Abbreviations: MS/MS, tandem mass spectrometry; Mw, molecular weight; OSCC, oral squamous cell carcinoma; OSF, oral submucosa fibrosis; pI, isoelectric point

a The *m/z* value was from the peak statistic results

b After MS/MS and MASCOT analysis, the peptide sequence was calculated

c The uniprot number, protein description and gene were get from the uniprot database

d Mw and theoretical pI was calculated by the EXPASY Compute pI/Mw tool

e The protein expression level in OSCC and OSF was compared according to the ion intensity
