# Supplementary material for: LRP6 is identified as a potential prognostic marker for oral squamous cell carcinoma via MALDI-IMS
Source: Cell Death Dis. 2017 Sep 7;8(9):e3035–. doi: 10.1038/cddis.2017.433 (PMC5636978; doi:10.1038/cddis.2017.433)
Supplement: Supplementary Information [file cddis2017433x1.docx]

**Supplementary infos**

**1. Materials and methods**

***1.1 Patients and specimens***

Ten OSCC specimens containing adjacent noncancerous areas for MALDI-IMS analysis and twenty normal oral mucous tissues for IHC analysis were collected from the department of oral and maxillofacial surgery, Hospital of Stomatology, Sichuan University. OSCC tissues were snap-frozen after surgery and stored at -150℃, prepared for MALDI-IMS analysis. None of the patients received chemotherapy, radiotherapy, other interventional palliative or therapeutic treatment prior to sampling. In addition, 28 OSCC tissues for IHC analysis were collected from the department of pathology, Hospital of Stomatology, Sichuan University. These OSCC tissues, as well as 20 normal tissues were paraffin-embedded, and were used for immunohistochemical analysis. Clinicopathologic stages of all the tumor specimens were determined by two experienced pathologists according to the TNM classification criteria defined by the American Joint Committee on Cancer (AJCC) [^1^](#_ENREF_1). All the samples were obtained with informed consent of patients. This study was approved by the Institutional Ethics Committee of Sichuan University.

***1.2 MALDI-IMS***

MALDI-IMS measurement was performed as previously reported with minor modifications [^2^](#_ENREF_2). The principle and workflow was described schematically in Fig. 1A.

*1.2.1 Sample Preparation*

The collected tissues were frozen in the liquid N_2_, and then the frozen sample was crysectioned into 10 µm thick sections without OCT covering (Leica CM 1900 cryostat Leica Biosystems, Nussloch, Germany) at -20°C. The consecutive serial section was placed onto conductive indium-tin-oxide (ITO) coated glass slides (Bruker Daltonics, Bremen, Germany) adapted for MALDI-IMS. Another adjacent slice was covered onto a glass slide and stained with hematoxylin and eosin (H&E). The HE staining slice was identified by two pathologists independently, and the tissue slice will be carefully discussed if the results were different. The cancerous and non-cancerous areas were identified, and these ITO slides selected for MALDI-IMS analysis were according to the HE results. The selected ITO slide was air-dried and then washed with ethanol solution (twice at 70% for 1 min each and once at 96% for 30 s). After ethanol washing, the slide was dried in bench vacuum desiccator for 1 h before matrix deposition. The 2,5-Dihydroxybenzoic acid (DHB, Bruker Germany) matrix was elucidated in 50%acetonitrile/0.2%trifluoroacetic acid with the concentration of 30 mg/ml. The matrix should be dissolved completely for the high efficient spraying. The matrix was sprayed according to the manufacturer’s introduction under the protection of N_2_ gas (ImagePrep, Bruker Daltonik GmbH, Bremen, Germany).

*1.2.2 MALDI Imaging*

Before MALDI image analysis, the mass spectrum was calibrated by the standard sample (Bruker). The standard sample was tipped onto the anchor chip plate, and then the DHB matrix was added. Calibration was progressed according to the standard program. The slide was scanned with a flatbed scanner and then conducted into the Ultraflex TOF/TOF mass spectrometer controlled by the FlexControl 3.3 software (Bruker Daltonics) in positive linear ion mode. The region for MALDI analysis was manually defined on the FlexImaging software through spatially relating the digital image to histological structures of their corresponding sister slides. The laser spot diameter was set with 50 µm and a raster size with 100 µm. Mass spectra were collected automatically over the mass range of 800-10,000 Da at 0.13 GS/s sampling rate across the entire tissue area. The MALDI-MIS results were collected and visualized by FlexImaging software, version 2.0 (Bruker Daltonics). The sister slides were stained with H&E and scanned with an automated slide scanner (Scanscope, Aperio, USA).

*1.2.3 Data acquisition and MS/MS analyses*

The regions of interest (ROIs) (OSCC as one group and corresponding non-tumor areas as another group for comparing the average peak intensity) were selected based on the H&E staining image and confirmed by an oral pathologist. ROIs from different tissues were loaded to Clinprot tools for further analysis. The total spectra of ten OSCC ROIs was 2306, and total spectra of ten non-tumor ROIs was 1879. ROIs including OSCC spectra was classified into one group, and ROIs including non-tumor spectra was classified into another group. The acquired mass spectra (OSCC and corresponding none-tumor areas) was calibrated on common “background” peaks (spectral alignment and matrix background peaks) and normalized to TIC (total ion count) algorithm utilizing ClinProTools 3.0 software (Bruker Daltonik) [^3^](#_ENREF_3). Wilcoxon rank-sum test was used to estimate the significant differences in peak intensities of ROIs between two different histological areas. The significance cutoff was set to 0.01. The results were corrected by Benjamini-Hochberg for multiple testing on a single data set and principle component analysis (PCA). Peaks with high quantity were chosen for further analyses (relatively higher S/N and intensity).

Followed by ClinProTools analyses, the MS/MS identification was conducted on the corresponding matrix-sprayed sister slide within the area with high detection of an individual peak. In the LIFT mode, the selected peaks were added to MS/MS list and each peak was solely set as the parent ion. The MS/MS results were confirmed with BioTools and MASCOT (Matrix Science, a local mascot library).

The spectrum was analyze by BioTools and MS/MS search was performed as the follows: the taxonomy was Homo sapiens the significance threshold of 0.05, error tolerance of 0.5 Da as well as no enzyme cleavage [^4^](#_ENREF_4). For each peptide, a calculated m/z value, mass number, a rank, a max score, and an E-value were given. The genetic information of proteins which were calculated with highest score by MS/MS analysis was processed for bioinformatics analysis in the context of molecular networks and to elucidate the complex physiological role of the proteins.

***1.3 Bioinformatics analysis***

The protein-protein interaction (PPI) network was applied to collect the biological evidence of the significant differentially expressed proteins identified by MS/MS [^5^](#_ENREF_5). Subsequently, network related proteins were grouped into 10 functional synaptic protein groups based on Gene Ontology (GO) Annotation clustering, which was performed using DAVID database (<http://david.abcc.ncifcrf.gov/>) [^6^](#_ENREF_6).

The represented functional groups containing more than 100 proteins were considered with relevant enrichment. The cell proliferation subnetworks were then assigned and Cytoscape was used to integrate the unified conceptual framework of PPI network.

***1.3.1 Function clustering and pathway analysis***

DAVID functional annotation tool was used to determine the functional enrichment [^7^](#_ENREF_7). Functional categories were performed using GO term related to Biological Process (BP), as well as pathway annotations derived from Kyoto encyclopedia of genes and genomes (KEGG) [^8^](#_ENREF_8)^,^ [^9^](#_ENREF_9).The obtained results of differentially expressed proteins which were in the form of UniProt accession numbers were analyzed by the gene ontology tool (GOTERMCCALL). Fisher’s exact test was applied and P < 0.05 yielded by GO terms was considered with significant enrichment in each gene list [^10^](#_ENREF_10). Pathways were considered significant with a corrected P < 0.05, and classified into hierarchical categories according to KEGG.

***1.4 Immunohistochemistry and Immunofluorescence***

Immunohistochemical studies were performed as a previous report described [^11^](#_ENREF_11). Briefly, sections were deparaffinized in xylene and rehydrated in graded ethanol: 100%, 95%, 80%, 75%, the following incubation with 3% H_2_O_2_ was performed in the dark at room temperature for 5 mins to quench the endogenous peroxidase activity. Antigens retrieval was performed with 0.01 M Ethylene Diamine Tetraacetic Acid buffer (EDTA) at 95°C. Sections were then preincubated in PBS containing 5% BSA for 30 min to block nonspecific epitopes. Subsequently, sections were incubated overnight at 4°C with primary antibody (anti-LRP6, ab118490, 1/50, Abcam; anti-FGF8, ab89550, 1/50, Abcam), followed by incubations with biotinylated secondary antibody for 1 h at 37°C. Finally, sections were visualized with 2, 3-diaminobenzidine (DAB) substrate solution and counter staining with Mayer’s hematoxylin. For immunofluorescence analysis, after overnight incubation with primary antibody (LRP6+FGF8), the tissue slices were washed with PBS three times. Then, the slices were incubated with secondary antibody with green or red fluorescence for 1h at 37°C. After washed three times with PBS, the slices were stained with DAPI and observed on a microscope (Olympus).

Cells with visible brown particles in the cytoplasm or cytomembrane were considered as positive cells. Results were assessed and confirmed independently by two experienced clinical pathologist，blinded to patient outcomes and the clinical parameters. For evaluating the immunostaining, more than eight individual fields in one slide were chosen, and 100 cells were counted per field. The score for each slide was measured based on the intensity and density of positive cells. Immunostaining intensity was divided into five grades: 0, negative; 1, weak; 2, moderate; 3, strong; 4, very strong. The density of staining-positive cells was also divided into five grades: 0, <5%; 1, 6-25%; 2, 26-50%; 3, 51-75%; 4, >75%. The final scores of each sample were the grade of immunostaining intensity multiplied by the grade of density, and the results were defined as: 0-4, low; 5-16, high.

***1.5 Quantitative real-time PCR***

HSC-3 and HSC-4 cells were obtained from cell bank of Japanese Collection of Research Bioresource (JCRB, Shinjuku, Japan), and cultured in DMEM (Gibco, Eggenstein, Germany), supplemented with 10% fetal bovine serum (FBS; Gibco) under 5% CO_2_ and humidified air atmosphere at 37°C.

Total RNA was extracted from cultured cells by using Trizol reagent (Invitrogen, Carlsbad, CA) and reversed transcribed by using PrimeScript RT reagent kit (Takara) following the manufacturer’s protocol. Quantitative real-time PCR was done using the SYBR Premix Ex Taq II kit (Takara) on a 7300 real-time PCR system (Applied Biosystems). The primers used for PCR amplification of FGF8 were: sense 5'-CAC TTG CTG GTC CTC TGC CTC CAA G-3' and antisense 5-GTA GTT GAG GAA CTC GAA GCG-3' [^12^](#_ENREF_12). LRP6: sense 5′-GAG CTG GAC TGT TAT CCA ACTG-3′ and anti-sense 5′-CTT CAT ACG AGG ACA CAG CATC-3′; Actin: sense: 5’-CAC GAT GGA GGG GCC GGA CTC ATC-3’ and antisense: 5’-TAA AGA CCT CTA TGC CAA CAC AGT-3’.

***1.6 Proliferation ability assays***

The CCK8 and colony formation assays were conducted as previously reported [^13^](#_ENREF_13). Notably, the HSC-3 and Cal-27 cells were transfected with siLRP6 or siNC, the CCK8 assay was conducted in the following 24h,48h,72h and the OD450 was valued. Colony formation assay was conducted as the following steps: after 48h transfection, 500 OSCC cells were seeded in the six well plate and cultured DMEM (Gibco) with 10% fetal bovine serum (Gibco, 37°C, 5% CO_2_) for ten days, the cells were stained with crystal violet and the spots were counted. As shown in Fig. 7, the HSC-3 and HSC-4 cell lines were transfected with vector, LRP6, LRP6+siNC, LRP6+siFGF8-1, LRP6+siFGF8-2, and the proliferation abilities were detected by CCK8 and colony formation assay as described.

***1.7 SiRNA transfection and immunoblot***

The siRNAs were transfected according to the standard manufacturers (ROCHE) and the siRNA targeting sequences were shown as follows, LRP6: 5'-3' CCG CAT GGT GAT TGA TGAA and ACA TTG TTC TGC AGT TAGC; FGF8: 5'-3' CTG TTC AGT CCT CAC CTAA and TGC TGG AGA ACA ACT ACAC. The immunoblot was conducted as previously reported [^13^](#_ENREF_13), primary antibodies used as follows: anti-LRP6, ab118490; anti-GAPDH, ab8245; anti-FGF8, ab89550.

***1.8 Statistical Analyses***

The quantitative variables was analyzed by Student’s t-test or one-way ANOVA when appropriate; Wilcoxon test was applied to compare categorical variables; Patients’ survival curve was plotted using the Kaplan-Meier method, and the log-rank test was used to determine the significant difference among groups. Pearson’s correlation coefficients were applied to determine the relation between two potential biomarkers (LRP6 and FGF8) over all cases. For all tests, P< 0.05 was considered statistically significant and the analyses were performed by SPSS 19.0 for Windows (SPSS, Chicago, IL).

1. Sobin LH, Compton CC. TNM seventh edition: What's new, what's changed. *Cancer* 2010;**116**: 5336-9.

2. Wisztorski M, Franck J, Salzet M, Fournier I. MALDI direct analysis and imaging of frozen versus FFPE tissues: what strategy for which sample? *Mass Spectrometry Imaging: Principles and Protocols* 2010: 303-22.

3. Huber K, Aichler M, Sun N, Buck A, Li Z, Fernandez I, Hauck S, Zitzelsberger H, Eickelberg O, Janssen K. A rapid ex vivo tissue model for optimising drug detection and ionisation in MALDI imaging studies. *Histochemistry and cell biology* 2014;**142**: 361-71.

4. Rotunno MS, Auclair JR, Maniatis S, Shaffer SA, Agar J, Bosco DA. Identification of a misfolded region in superoxide dismutase 1 that is exposed in amyotrophic lateral sclerosis. *Journal of Biological Chemistry* 2014;**289**: 28527-38.

5. Zhang QC, Petrey D, Garzón JI, Deng L, Honig B. PrePPI: a structure-informed database of protein–protein interactions. *Nucleic acids research* 2013;**41**: D828-D33.

6. Huang DW, Sherman BT, Lempicki RA. Bioinformatics enrichment tools: paths toward the comprehensive functional analysis of large gene lists. *Nucleic acids research* 2009;**37**: 1-13.

7. Huang DW, Sherman BT, Lempicki RA. Systematic and integrative analysis of large gene lists using DAVID bioinformatics resources. *Nature protocols* 2009;**4**: 44-57.

8. Consortium GO. The Gene Ontology (GO) project in 2006 Nucleic Acids Res,. 34. *D322CD326* 2006.

9. Kanehisa M, Goto S. KEGG: kyoto encyclopedia of genes and genomes. *Nucleic acids research* 2000;**28**: 27-30.

10. Du J, Yuan Z, Ma Z, Song J, Xie X, Chen Y. KEGG-PATH: Kyoto encyclopedia of genes and genomes-based pathway analysis using a path analysis model. *Molecular bioSystems* 2014;**10**: 2441-7.

11. Liu R, Li J, Xie K, Zhang T, Lei Y, Chen Y, Zhang L, Huang K, Wang K, Wu H. FGFR4 promotes stroma-induced epithelial-to-mesenchymal transition in colorectal cancer. *Cancer research* 2013;**73**: 5926-35.

12. Marsh S, Bansal G, Zammit C, Barnard R, Coope R, Roberts-Clarke D, Gomm J, Coombes R, Johnston C. Increased expression of fibroblast growth factor 8 in human breast cancer. *Oncogene* 1999;**18**: 1053-60.

13. Yuan Y, Hou X, Feng H, Liu R, Xu H, Gong W, Deng J, Sun C, Gao Y, Peng J, Wu Y, Li J, et al. Proteomic identification of cyclophilin A as a potential biomarker and therapeutic target in oral submucous fibrosis. *Oncotarget* 2016.
