# Supplementary material for: LRP6 is identified as a potential prognostic marker for oral squamous cell carcinoma via MALDI-IMS
Source: Cell Death Dis. 2017 Sep 7;8(9):e3035–. doi: 10.1038/cddis.2017.433 (PMC5636978; doi:10.1038/cddis.2017.433)
Supplement: Supplementary Table S1 [file cddis2017433x2.docx]

**Supplementary Table S1 Altered peaks identified by MALDI-IMS**

| Peak No | m/z | Alteration(  OSCC/Normal) | Mean Intensity  in OSCC (a.u.) | Mean Intensity  in Normal (a.u.) |
| --- | --- | --- | --- | --- |
| 1 | 5322.37 | ↑ | 2.58 | 1.20 |
| 2 | 4275.95 | ↑ | 3.68 | 1.80 |
| 3 | 4089.27 | ↑ | 3.04 | 1.49 |
| 4 | 3409.33 | ↑ | 2.79 | 1.39 |
| 5 | 3481.53 | ↑ | 3.69 | 1.86 |
| 6 | 3525.52 | ↑ | 2.67 | 1.36 |
| 7 | 823.27 | ↑ | 9.87 | 5.87 |
| 8 | 3424.76 | ↑ | 2.67 | 1.63 |
| 9 | 4419.02 | ↑ | 2.44 | 1.49 |
| 10 | 5462.65 | ↑ | 1.88 | 1.16 |
| 11 | 3315.20 | ↑ | 2.44 | 1.56 |
| 12 | 9860.72 | ↑ | 1.02 | 0.70 |
| 13 | 4358.51 | ↑ | 1.86 | 1.29 |
| 14 | 3961.90 | ↑ | 2.02 | 1.42 |
| 15 | 8618.08 | ↑ | 1.34 | 0.95 |
| 16 | 6250.85 | ↑ | 1.27 | 0.94 |
| 17 | 4970.09 | ↑ | 1.58 | 1.17 |
| 18 | 1787.81 | ↑ | 2.39 | 1.77 |
| 19 | 5102.87 | ↑ | 1.93 | 1.43 |
| 20 | 3364.50 | ↑ | 2.61 | 1.94 |
| 21 | 6040.00 | ↑ | 1.21 | 0.91 |
| 22 | 3013.53 | ↑ | 2.96 | 2.23 |
| 23 | 5379.04 | ↑ | 1.48 | 1.12 |
| 24 | 5263.01 | ↑ | 1.36 | 1.04 |
| 25 | 2192.06 | ↑ | 2.16 | 1.68 |
| 26 | 5891.30 | ↑ | 1.29 | 1.02 |
| 27 | 5034.58 | ↑ | 1.51 | 1.20 |
| 28 | 5055.07 | ↑ | 1.42 | 1.13 |
| 29 | 4996.49 | ↑ | 2.07 | 1.65 |
| 30 | 7507.34 | ↑ | 1.02 | 0.82 |
| 31 | 5772.02 | ↑ | 1.22 | 0.99 |
| 32 | 4673.57 | ↑ | 1.93 | 1.57 |
| 33 | 7337.49 | ↑ | 1.00 | 0.83 |
| 34 | 7291.65 | ↑ | 1.00 | 0.83 |
| 35 | 6918.53 | ↑ | 0.99 | 0.84 |
| 36 | 7975.06 | ↑ | 0.95 | 0.81 |
| 37 | 2380.63 | ↑ | 2.36 | 2.02 |
| 38 | 8282.71 | ↑ | 0.90 | 0.79 |
| 39 | 5016.24 | ↑ | 1.39 | 1.23 |
| 40 | 7697.85 | ↑ | 0.88 | 0.78 |
| 41 | 8398.86 | ↑ | 0.82 | 0.73 |
| 42 | 941.89 | ↑ | 3.50 | 3.21 |
| 43 | 9059.75 | ↑ | 0.84 | 0.81 |
| 44 | 8750.70 | ↑ | 0.91 | 0.88 |
| 45 | 4806.43 | ↑ | 1.57 | 1.52 |
| 46 | 4622.59 | ↓ | 2.85 | 2.90 |
| 47 | 8191.75 | ↓ | 0.86 | 0.88 |
| 48 | 7654.03 | ↓ | 0.91 | 0.95 |
| 49 | 8660.13 | ↓ | 0.94 | 1.02 |
| 50 | 1326.66 | ↓ | 1.99 | 2.18 |
| 51 | 890.15 | ↓ | 3.48 | 3.86 |
| 52 | 8548.44 | ↓ | 2.54 | 2.84 |
| 53 | 947.52 | ↓ | 3.67 | 4.19 |
| 54 | 1156.78 | ↓ | 2.02 | 2.32 |
| 55 | 3585.28 | ↓ | 1.81 | 2.09 |
| 56 | 907.97 | ↓ | 2.75 | 3.21 |
| 57 | 931.02 | ↓ | 3.64 | 4.26 |
| 58 | 979.48 | ↓ | 2.04 | 2.39 |
| 59 | 1149.79 | ↓ | 1.80 | 2.14 |
| 60 | 1617.45 | ↓ | 1.95 | 2.35 |
| 61 | 1592.30 | ↓ | 2.80 | 3.56 |
| 62 | 4211.17 | ↓ | 1.72 | 2.20 |
| 63 | 1565.83 | ↓ | 3.28 | 4.20 |
| 64 | 1512.10 | ↓ | 2.29 | 3.19 |
| 65 | 1538.99 | ↓ | 2.97 | 4.15 |
| 66 | 924.83 | ↓ | 2.99 | 4.33 |
| 67 | 808.69 | ↓ | 2.98 | 7.23 |

a.u. arbitrary unit
