# Supplementary material for: LRP6 is identified as a potential prognostic marker for oral squamous cell carcinoma via MALDI-IMS
Source: Cell Death Dis. 2017 Sep 7;8(9):e3035–. doi: 10.1038/cddis.2017.433 (PMC5636978; doi:10.1038/cddis.2017.433)
Supplement: Supplementary Table S2 [file cddis2017433x3.docx]

**Supplementary Table S2 Relations between LRP6 expression and clinicopathologic parameters**

| Group | Number | LRP6 | |  |
| --- | --- | --- | --- | --- |
|  |  | Mean | Standard deviation | *P* value |
| Gender |  |  |  | 0.1218 |
| Male | 19 | 11.58 | 3.40 |  |
| Female | 9 | 9.44 | 3.05 |  |
| Age | |  |  | 0.1554 |
| ≤40 | 3 | 13.67 | 4.04 |  |
| 40-60 | 10 | 9.40 | 2.88 |  |
| ＞60 | 15 | 11.33 | 3.33 |  |
| Smoking |  |  |  | 0.0047** |
| Yes | 8 | 13.63 | 2.72 |  |
| No | 20 | 9.80 | 3.04 |  |
| Drinking |  |  |  | 0.0095** |
| Yes | 13 | 12.62 | 3.45 |  |
| No | 15 | 9.40 | 2.61 |  |
| lesion site | |  |  | 0.6646 |
| Tongue | 18 | 10.33 | 3.73 |  |
| Bucca | 5 | 12.20 | 2.49 |  |
| Floor of mouth | 2 | 12.50 | 4.95 |  |
| Gingiva | 3 | 11.00 | 1.73 |  |
| Tumor size | |  |  | 0.5202 |
| ≤2 | 9 | 10.56 | 3.64 |  |
| ＞2，≤4 | 14 | 11.57 | 3.18 |  |
| ＞4 | 5 | 9.60 | 3.78 |  |
| Histological degree | |  |  | 0.0056** |
| Well differentiation | 18 | 9.50 | 2.46 |  |
| Moderate differentiation | 5 | 13.20 | 3.83 |  |
| Poor differentiation | 5 | 13.80 | 3.19 |  |
| Clinical stage |  |  |  | 0.1656 |
| I | 9 | 9.44 | 2.92 |  |
| II | 13 | 11.00 | 3.19 |  |
| III | 6 | 12.83 | 3.71 |  |
| Lymph node metastasis | |  |  | 0.0206* |
| Yes | 9 | 13.00 | 2.45 |  |
| No | 19 | 9.89 | 3.36 |  |
| TNM stage |  |  |  | 0.0655 |
| I/II | 16 | 12.25 | 3.36 |  |
| III/Ⅳ | 12 | 9.88 | 3.14 |  |

**P* < 0.05 statistically significant
